# Fabrication of Nanostructures on Surface of Micro-Lens Arrays Using Reactive Ion Etching

**DOI:** 10.3390/mi16121306

**Published:** 2025-11-21

**Authors:** Tae Jeong Hwang, Eun Jeong Bae, Geun-Su Choi, Young Wook Park

**Affiliations:** 1 Nano and Organic-Electronics Laboratory, Department of Display and Semiconductor Engineering, Sun Moon University, Asan 31460, Republic of Korea; zeratull1234@sunmoon.ac.kr (T.J.H.); baeejng@korea.ac.kr (E.J.B.); crs4964@korea.ac.kr (G.-S.C.); 2 Display and Nanosystem Laboratory, Department of Electrical Engineering, Korea University, Seoul 02841, Republic of Korea; 3 Center for Next Generation Semiconductor Technology, Department of Display and Semiconductor Engineering, Sun Moon University, Asan 31460, Republic of Korea

**Keywords:** organic light-emitting diodes, micro-lens array, reactive ion-etching, external light extraction, nanostructure, nanostructure distribution

## Abstract

In this study, we fabricated a nanostructure on the surface of the micro-lens array (MLA), which is one of the light extraction technologies of organic light-emitting diodes (OLEDs), by performing the Reactive Ion -Etching (RIE) process. The MLA consists of a lensed area and a lens-less bottom (flat film area). We performed a systematic analysis to find ways to improve the light extraction efficiency of the MLA surface and flat film area. By controlling the RIE process time and type of gas plasma, nanostructures were formed on the surface of the MLA. O_2_ and CF_4_ gas plasmas resulted in nanostructures with tall heights and high aspect ratios, whereas CHF_3_ and Ar gas plasmas resulted in nanostructures with small heights and low aspect ratios. Furthermore, it was found that the nanostructures were not covered over the entire area, and the extent to which the nanostructures were distributed varied depending on the process time. As the RIE process time increases, the nanostructure expands from the top surface of the MLA to the flat film area. This limited the light extraction efficiency improvement. At a short process time of 50 s, nanostructures were formed only on the upper surface of the MLA hemisphere, which increased the light extraction efficiency. However, at long process times over 50 s, the surface of the hemisphere of MLA was covered with vertically aligned nanostructures, which decreased the efficiency. While the flat film area was covered with nanostructures at the longest process time of ~3200 s, it was effective, but the total efficiency was further decreased by the trade-off between them. As a result, the high-aspect-ratio nanostructured MLA patterned only on the top surface of the hemispherical MLA with a 50 s O_2_ plasma treatment showed the highest efficiency, which was slightly higher than that of the bare MLA. We expect that if the nanostructures can be formed in a direction perpendicular to the MLA surface and the flat film area simultaneously, the light extraction efficiency would be further improved.

## 1. Introduction

Organic light-emitting diodes (OLEDs) are used in a variety of fields, including flat panel displays and lighting, because they have advantages such as thinness, self-emission, low power consumption, and flexible properties [1,2,3,4]. However, OLEDs still have a challenge to overcome, which is the optical loss. The internal quantum efficiency of OLEDs has reached 100%, but in conventional OLED structures, the light emitted outside is limited due to the optical loss occurring in the surface plasmon polariton (SPP), substrate mode, and waveguide mode by the refractive index difference between thin layers [5,6]. Therefore, various light extraction technologies have been studied to implement high-efficiency OLEDs. Internal light extraction techniques to improve the waveguide mode and SPP include microcavity structures, low-index grid structures, and scattering layers, which are difficult to implement because they require changes in the thickness and structure of the device [7,8,9,10,11,12,13,14,15,16,17]. On the other hand, external light extraction technology does not require any change in the structure of the device, so it can be simply applied to the device to extract the light trapped inside the substrate to the outside, thereby increasing efficiency. For example, one of the most commonly used technologies is the micro-lens array (MLA) [18,19,20,21]. Generally, an MLA is a structure in which hemispherical lenses with a diameter of dozens of micrometers are arranged. When applied to the outside of an OLED substrate, it reduces the incident angle at the interface between the hemispherical lens surface and the outside (air) and extracts most of the light that would otherwise be totally reflected at the interface between the substrate and the outside (air), greatly improving the light extraction efficiency. As an external light extraction technology that does not affect the structure of the OLED device, it can extract more light efficiently. However, it is hard to extract all the light with the MLA, since there is incident light on the lens surface at an angle over the critical angle, and also there is the incident light in the flat film lens-less area. Thus, to increase the light extraction efficiency, it is important to control this. To solve these problems, a method is needed to reduce the incidence angle of light with extreme angles on the lens surface while maintaining existing light extraction and to improve the low light extraction efficiency of flat film regions. In this study, we used a high-aspect-ratio nanostructuring method to improve efficiency while maintaining the structural characteristics of the MLA. By forming nanostructures with a uniform size and density, we maintained the existing light extraction characteristics and reduced the incidence angle of light at extreme angles. Furthermore, by forming flat film regions with this structure, we were able to improve the light extraction efficiency of these regions. Methods for forming such surface nanostructures include photolithography, imprinting, and naturally formed patterns. However, photolithography and imprinting have the disadvantages of high process costs and the difficulty of fabricating large areas. In this study, we systematically studied the formation and structural properties of nanostructures using naturally formed nanostructures in the RIE dry etching process, a low-cost process that allows for easy large-area fabrication. Therefore, in this study, the formation and structural characteristics of nanostructures were investigated in detail step by step. To clarify the nanostructure formation mechanism in detail and simplify the fabrication process, the nanostructure formation process by single gas (O_2_, CHF_3_, Ar, and CF_4_) plasma was analyzed in detail. It can be confirmed that the shape of the nanostructure changed depending on the gas plasma and RIE process time. As the RIE process time increased, the nanostructure formed first from the top of the hemisphere MLA and went to the bottom side and the flat area. Also, the direction of the nanostructure was not isotropic to the sphere surface but upward to the substrate. This formation characteristic was found to be a limitation of the light extraction characteristics. We predict that the formation of a uniform nanostructure with isotropic directional growth on the flat film area and the hemisphere surface would increase the light extraction efficiency, and further investigation would be possible.

## 2. Experimental Details

### 2.1. Fabrication of Nanostructured Hybrid MLA

**Figure 1:**
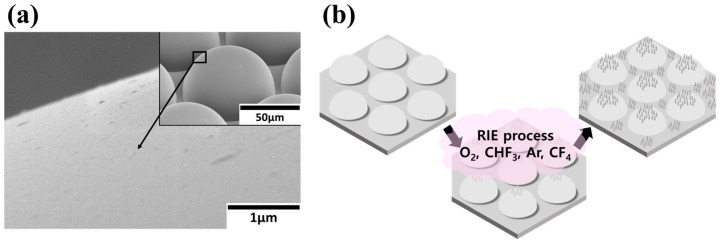
(**a**) A 45° tilted FE-SEM image of the bare MLA. (**b**) A schematic of the RIE process for fabricating the nanostructured MLA.

The RIE process was performed on the MLA surface. In Figure 1a, the field emission scanning electron microscope (FE-SEM) image of the MLA surface used in the fabrication process is presented. In the inset image of Figure 1a, the commercial MLA (OCA, MNtech Co., Ltd., Cheongju-si, Replublic of Korea) which is based on a PET film, with a diameter of 75 µm, a fill factor of 5 µm, and a hemispherical pattern structure filled with hexagons—is used. Figure 1b shows the fabrication process of the nanostructured MLA. The nanostructures were formed on the surface of the MLA, using the RIE process through various gas plasmas of O_2_, CHF_3_, Ar, and CF_4_. RIE was performed at 32 mTorr, 30 sccm, and 200 W. The RIE process is a standardized dry etching process widely used in semiconductor manufacturing and has excellent process reproducibility and uniformity. When polymer etching is performed with F-series gases, organic molecules bonded with F radicals and ions are generated, and when these molecules clump together, aggregates are formed whose surfaces are surrounded by F. The surface surrounded by these F functional groups prevents bonding with O radicals and ions, lowering the etching rate of the relevant portion, acting as an etching mask [22]. And this difference in partial etching rates causes the formation of vertical nanostructures. To control the shape and size of the nanostructures, the RIE process time was adjusted from 12.5 s to 400 s. The RIE process time and type of gas affected the height, width, and shape of the nanostructures.

### 2.2. Fabrication of Organic Light-Emitting Diodes

A 1-inch soda-lime glass coated with 185 nm of the indium tin oxide (ITO) was cleaned by ultrasonication with acetone, methanol, and deionized water, subsequently for 15 min each. Using a photoresist (AZ 601 GXR, AZ Electronic Materials Co., Ltd., Darmstadt, Germany) via photolithography, a 6.25 mm diameter circular active area of the OLED was defined. The ITO surface was treated with UV-ozone (UVC-300, Omniscience, Yongin-si, Republic of Korea) and O_2_ plasma (CUTE, Femto Science Co., Hwaseong-si, Republic of Korea) for work function adjustment and the removal of residual contaminants. The substrates were rotated at 12 rpm during the thermal evaporation process. The green fluorescent OLEDs were deposited under high vacuum (~10^−7^ Torr) and structured as follows: 60 nm of N, N′-Bis(naphthalen-1-yl)-N, N′-bis(phenyl)-benzidine (NPB) as the hole transport layer, 60 nm of tris(8-hydroxyquinolinato)-aluminum (Alq_3_) as the emission and the electron transport layer, 1 nm of lithium fluoride (LiF) as the electron injection layer, and 100 nm aluminum (Al) as the cathode.

### 2.3. Electroluminescence Characterization and Measurement

The aspect ratio of the nanostructures was measured at a 45° tilt using an FE-SEM (Helios G4, ThermoFisher Scientific, Waltham, MA, USA; resolution < 0.7 nm). To analyze the nanostructured MLA, an optically clear adhesive (OCA, MNtech Co., Ltd., Cheongju-si, Replublic of Korea) film was attached to the bottom of the substrate. The viewing angle and electroluminescence (EL) characteristics were measured using a spectroradiometer (CS-2000A, Konica Minolta Co., Ltd., Chiyoda, Tokyo, Japan) and a source meter (Keithley-2400, Tektronix, Beaverton, OR, USA). By using vacuum (~10^−3^ Torr) jigs and fully automated XYZR-axis equipment, the viewing angle characteristics were measured by rotating OLEDs in 5° increments from 0° to 70°. To measure the EL characteristics, including power efficiency, current efficiency, and EQE, the aperture of the spectroradiometer was set to 1°, which corresponds to the 5 mm diameter of the actually measured circle at the 0° axis. In the viewing angle measurement, the aperture was set to 0.2°, which corresponds to the 1 mm diameter of the actual measured circle at 0° axis and 2.92 mm diameter of the actual measured ellipse at 70° axis. The power efficiency and EQE were simply calculated by assuming Lambertian emissions and then corrected using viewing angle characteristics, including actual measured and projected areas.

## 3. Results and Discussion

### 3.1. Nanostructure Characteristics

**Figure 2:**
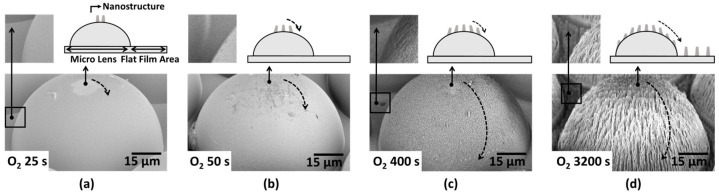
(**a**–**d**) Nanostructure formation changes by RIE process time (O_2_ gas plasma).

**Figure 3:**
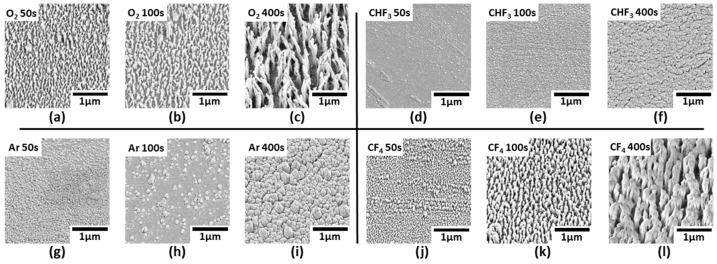
FE-SEM images of the fabricated nanostructures on top surface of MLA by the different gas types and RIE process time. (**a**–**c**) O_2_ gas plasma, (**d**–**f**) CHF_3_ gas plasma, (**g**–**i**) Ar gas plasma, (**j**–**l**) CF_4_ gas plasma.

**Figure 4:**
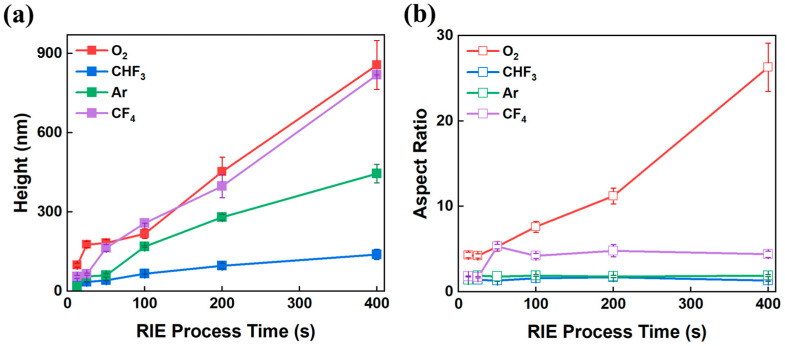
(**a**) RIE process time to height of nanostructure and (**b**) RIE process time to aspect ratio (height divided by the radius of the bottom surface) of nanostructure.

Figure 2 shows the FE-SEM images of the surface of the MLA with the treated O_2_ gas plasma according to the RIE process time increase. The FE-SEM images of MLAs at all other RIE process times, including those in Figure 2, are shown in Figure S1. O_2_ plasma etching is based on a chemical etching mechanism in which atomic oxygen reacts with the polymer surface, causing oxidation and chain scission. This reaction decomposes the polymer backbone, forming nanostructures. The volatile substances produced during this process (e.g., CO, CO_2_) are removed by a vacuum pumping system. The high reactivity of the O_2_ plasma allows for the formation of nanostructures with high etch rates and high aspect ratios compared to other gas plasmas [23,24,25]. Interestingly, the formation of nanostructures was not observed over the entire surface of the MLA. At a short process time of 25 s, the nanostructures were formed only at the MLA peak positions (Figure 2a), covering about ~10% of the total surface area of the hemisphere. When the process time increased to 400 s, the coverage of nanostructures increased overall (Figure 2b), and the nanostructure became larger and thicker as time increased (Figure 2c). Figure 3 shows FE-SEM images of the nanostructures on the surface of the MLA treated with the single gas plasma (O_2_, CHF_3_, Ar, and CF_4_) over time. The FE-SEM images of MLAs at all other RIE process times, including those in Figure 3, are shown in Figure S2. Figure 4 and Table 1 show the height and aspect ratio of the nanostructures over the RIE process time. The height and bottom radius of the nanostructures were measured using an FE-SEM. The aspect ratio was calculated by dividing the measured height by the measured radius. Performing the RIE process on the surface of the MLA, it can be confirmed that there is a difference between the height and aspect ratio depending on each gas plasma. In Figure 4a, all gas plasmas show an increase in the height of the nanostructures as the RIE process time increases. The accuracy of the measurements is reflected in the error bars shown in Figure 4, which represent the variability observed across multiple measurements. Notably, the height of nanostructures in O_2_ and CF_4_ gas plasmas is overall higher than that of the CHF_3_ and Ar gas plasmas. And the highest height at the same process time was 854 nm at 400 s for the O_2_ gas plasma. This corresponds to up to a 6.14-fold higher height than the nanostructure treated with the CHF_3_ gas plasma during the same process time at 400 s (139 nm, which has the minimum height among the listed values). In Figure 4b, the aspect ratio increased continuously only for the O_2_ gas plasma as the RIE process time increased, showing 26.3 at 400 s. This is up to 20.23-fold higher than the aspect ratio of the nanostructure treated with the CHF_3_ gas plasma during the same process time of 400 s (1.3, which has the minimum aspect ratio among the listed values). This is considered to be due to the high etching rate of the O_2_ gas plasma, the polymerization characteristics of the CF_4_ and CHF_3_ gas plasmas, and the low selectivity of the Ar gas plasma [26]. As a result, the height and aspect ratio were highest for the O_2_ gas plasma with a high etching rate, followed by the CF_4_ gas plasma.

### 3.2. EL Characteristics

**Figure 5:**
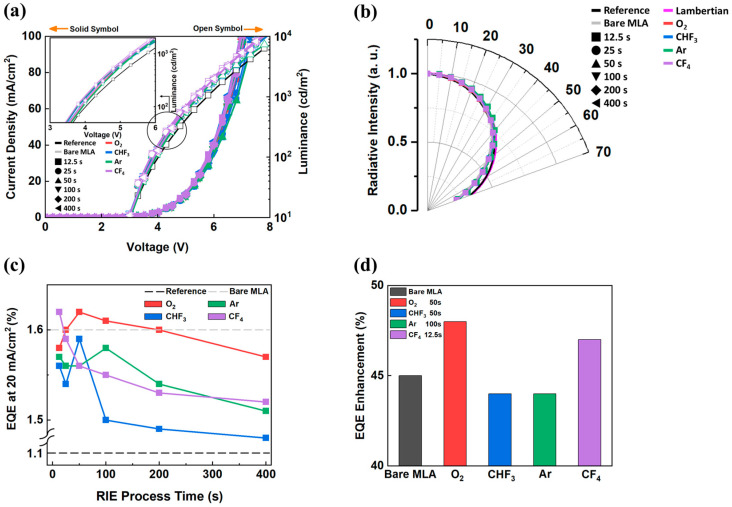
EL characteristics of samples (**a**) J-V-L curve, (**b**) viewing angle curve, (**c**) EQE of all samples at 20 mA/cm^2^, and (**d**) EQE enhancement at 20 mA/cm^2^ of selected samples compared to the reference.

**Figure 6:**
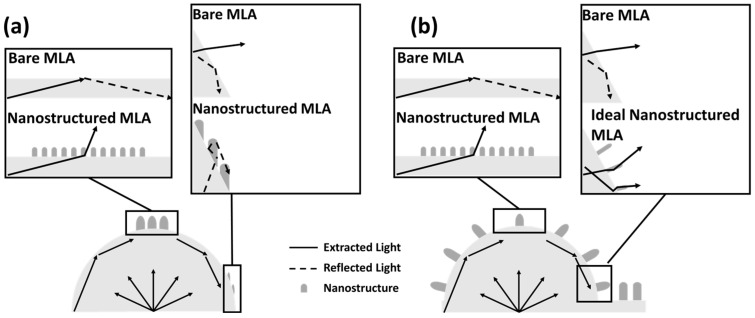
(**a**) A schematic of the nanostructured MLA. (**b**) A schematic of the ideal nanostructured MLA.

The nanostructured MLA was attached to the glass of bottom-emitting OLEDs using OCA, and the EL characteristics were measured. The reference is a bare OLED without an MLA. Figure 5a,b shows the EL characteristics of all samples. In Figure 5a, all the samples show almost identical current density–voltage characteristics as designed, since the OLEDs were fabricated identically to evaluate the external light extraction characteristics. And the luminance of all the samples shows a clear enhancement compared to the reference. Interestingly, the viewing angle characteristics in Figure 5b are almost identical. The fabricated nanostructures are rod-shaped, and although there are differences in the shape and smoothness depending on the process conditions, there is no significant difference in the viewing angle characteristics. This suggests that the shape difference is not the major factor here. The formed nanostructures were shown to be formed in a direction perpendicular to the substrate throughout the entire RIE process time range. This indicates that the nanorods formed in the perpendicular direction have an almost identical light distribution to the bare MLA. However, as the RIE process time increases, the nanostructures are formed not in the ideal spherical direction but rather are vertically aligned to the substrate. This leads to optical losses, as the vertical alignment of the nanostructures on the side surfaces of the MLA interferes with the light extraction, reducing the overall EQE and limiting the efficiency improvement compared to the bare MLA. A more detailed explanation is provided in Figure 6. In Figure 5c, the EQE at 20 mA/cm^2^ of all samples is presented and compared with the 1.1% EQE of the reference and the 1.6% EQE of the bare MLA. Detailed EL characteristics—including the J-EQE and current efficiency–luminance–power efficiency, following the type of gas plasma—are separated into each graph and are provided in Figures S3 and S4. And the EQE at 20 mA/cm^2^ for each condition is summarized in Table 2. The summarized highest EQE enhancement of each gas plasma is presented in Figure 5d. The 50 s O_2_ gas plasma sample showed the highest EQE of 1.63%, which is 1.48-fold higher than the reference. The 12.5 s CF_4_ gas plasma sample showed an increased EQE of 1.62%, which is 1.47-fold higher than the reference. The high EQE enhancement observed in the MLA treated with 50 s of O_s_ gas plasma, despite partial patterning, can be explained by the formation of vertically aligned nanostructures at the top surface of the MLA. These nanostructures, with a high aspect ratio, reduced optical losses, particularly at the top of the hemisphere, thereby enhancing light extraction efficiency and resulting in a higher EQE, even with limited coverage of the nanostructures, while the other two (CHF_3_ and Ar gas plasma) did not show as much EQE enhancement as the previous two gases (O_2_, CF_4_ gas plasma). The CHF_3_ and Ar gas plasma-treated MLA samples show an overall enhancement, even with a decreased EQE than the bare MLA, due to a low aspect ratio and smooth pattern resulting in an increased incident angle. The low-aspect-ratio nanostructures in the CHF_3_ gas plasma resulted from the condensation polymerization induced by fluorine. And the non-chemically reactive Ar gas plasma evenly etched polymers without directionality, resulting in surface planarization [27,28]. The O_2_ and CF_4_ gas plasmas act differently. The EQE of the MLA treated with O_2_ and CF_4_ gas plasma decreases when the RIE process time exceeds 200 s and 25 s, respectively, and becomes lower than that of the bare MLA. This can be explained by the complex effect of the structure height, aspect ratio, and coverage. Since the O_2_ gas plasma keeps increasing the height and aspect ratio following the RIE process time, the CF_4_ gas plasma shows the highest aspect ratio at 50 s. Similarly to the CHF_3_ gas plasma, the fluorine in the CF_4_ gas plasma induces aggregated polymerization and forms larger and wider structures with a limited aspect ratio, which is followed by a decrease in the EQE. And in O_2_ gas plasma samples, when the RIE process time increases, as in Figure 2, the coverage of the nanostructure increases but does not have a spherical direction and is not in the upward direction to the substrate, followed by reduced light extraction at the side bottom surface of the hemisphere.

At a short process time of ~50 s, the nanostructures formed only on the top surface of the hemisphere of the MLA, which led to an increase in the light extraction efficiency. However, at a long process time of over 50 s, all of the surface of the hemisphere of the MLA was covered with vertically aligned nanostructures, which decreased the efficiency. While the bottom flat lens-less area was covered with nanostructures at the longest process time of ~3200 s, it was effective, but the total efficiency was further decreased by the trade-off between them (Figure S5). It is expected that the EQE will be further improved as more nanostructures are formed perpendicular to the MLA side and in the flat film area. On the other hand, nanostructure implementation technology with an alignment direction perpendicular to the lens surface, similar to the one presented recently, has been reported, but much applied research is needed to directly utilize this in this study, as it requires the use of changes in the electric field distribution using conductors [29]. If further research can lead to the implementation of an ideal nanostructured MLA, this is expected to contribute to the implementation of high-efficiency light-emitting devices.

A comparison of light passages in upward-formed nanostructures and ideally spherical-formed nanostructures is shown in Figure 6. As the RIE process time increases, the nanostructure coverage on the sphere surface shifts from the top to the bottom (Figure 6a). The improved EQE of the nanostructures formed only on the top at short process times allows them to extract more high-incidence-angle light that the hemispheres cannot fully extract. However, at long process times, the nanostructures formed on the sides of the hemisphere are formed in a direction perpendicular to the substrate bottom. In this case, light that was successfully extracted before the nanostructures were formed may not be extracted due to the increased angle of incidence. Therefore, it is expected that more light will be extracted if the nanostructures are formed in a direction perpendicular to the sphere side rather than in a direction perpendicular to the substrate (Figure 6b).

## 4. Conclusions

In this study, we fabricated a nanostructured MLA using various gas plasmas through the RIE process on a conventional MLA and investigated the effect on the EL characteristics. We formed nanostructures of various sizes and shapes by controlling the single gas (O_2_, CHF_3_, Ar, and CF_4_) plasma and RIE process time. We found that a nanostructure shape on the surface of the MLA with a straight, high aspect ratio, rather than in a smooth nanostructure shape with a low aspect ratio, is more effective in the external extraction of light reflected inside the hemisphere. It was confirmed that the O_2_ and CF_4_ gas plasma-treated MLAs formed a high-aspect-ratio nanostructure of 26.3 and 5.3, respectively, and the CHF_3_ and Ar gas plasma-treated MLAs formed a low-aspect-ratio nanostructure. Specifically, the MLA attached device with a straight, sharp, and high-aspect-ratio nanostructure treated with 50 s of the O_2_ gas plasma showed the highest EQE enhancement of 48% compared to the reference.

While the EQE was slightly improved compared to the bare MLA, this study shows how nanostructure formation on the MLA surface varies depending on the gas plasma and RIE process time. This demonstrates that the height, aspect ratio, and coverage of nanostructures can be systematically controlled. This is confirmed to be oriented from the unideal orientation of the nanostructure formation on the MLA surface aligned in the direction of the gas plasma (vertical direction). If the nanostructures grow isotropically across the entire hemispherical surface and extend to the flat film area, rather than just vertically forming on the top of the MLA surface, a greater EQE improvement is expected.

In conclusion, we systematically investigated the possibility of improving the light extraction efficiency of MLAs. Utilizing the nanostructured surface on the MLA by one-step RIE, we found that the structure with a high height, sharpness, and a high aspect ratio was more effective. Furthermore, we analyzed the nanostructure growth on the surface of the MLA and discussed how the non-ideal vertical growth direction limits light extraction and how extending nanostructure formation to the flat film area can further enhance the EQE. These points clarify the process–structure–performance relationship on curved MLA surfaces, which has not been systematically addressed in previous reports. Although the improvement in the EQE by introducing nanostructures is limited, we have confirmed their potential to further enhance light extraction. We confirmed that when nanostructures are optimized and implemented, they can improve optical loss that cannot be improved by MLAs, namely the high-incidence-angle light reflected into the inside of the MLA and optical loss in the flat film region. To fulfill and realize this structure, the technology to form the optimized nanostructure is essential, as well as a simultaneous nanostructure forming process over lens and lens-less areas, and further investigation is expected.

## Figures and Tables

**Table 1:** Height/aspect ratio of the nanostructure.

Height (nm)/Aspect Ratio						
Gas/Time (s)	12.5	25	50	100	200	400
O_2_	99/4.3	177/4.2	182/5.3	218/7.6	453/11.2	856/26.3
CHF_3_	22/1.4	35/1.4	41/1.3	66/1.6	96/1.7	139/1.3
Ar	16/1.4	56/1.9	60/1.8	169/1.9	280/1.8	445/1.9
CF_4_	55/1.8	65/1.7	164/5.3	258/4.2	398/4.8	819/4.4

**Table 2:** EQE at 20 mA/cm^2^ (%).

EQE at 20 mA/cm^2^ (%) Reference: 1.10 Bare MLA: 1.60						
Gas/Time (s)	12.5	25	50	100	200	400
O_2_	1.58	1.60	1.63	1.61	1.6	1.57
CHF_3_	1.56	1.54	1.59	1.50	1.49	1.48
Ar	1.57	1.56	1.56	1.58	1.54	1.51
CF_4_	1.62	1.59	1.58	1.55	1.53	1.52

## Data Availability

The original contributions presented in this study are included in the article/Supplementary Material. Further inquiries can be directed to the corresponding author.

## Supplementary Materials

The following supporting information can be downloaded at https://www.mdpi.com/article/10.3390/mi16121306/s1: Figure S1: SEM images of the nanostructures formation change by RIE treatment time; Figure S2: SEM images of the nanostructures fabricated by the different gas plasma; Figure S3: The current density-EQE characteristics; and Figure S4: The luminance-current efficiency, luminance-power efficiency characteristics; Figure S5: (a) SEM images of the nanostructures formed at 3200 s in O_2_ gas plasma (b) EQE Enhancement compared to Reference.
