# Supplementary material for: Fabrication of Nanostructures on Surface of Micro-Lens Arrays Using Reactive Ion Etching
Source: Micromachines (Basel). 2025 Nov 21;16(12):1306. doi: 10.3390/mi16121306 (PMC12734499; doi:10.3390/mi16121306)
Supplement: Supplementary file 1 [file micromachines-16-01306-s001.zip › micromachines-3982596-supplementary.pdf]

Supplementary Materials

Fabrication of Nanostructures on Surface of Micro-Lens Arrays Using Reactive Ion Etching

Tae Jeong Hwang [1], Eun Jeong Bae [1,2], Geun-Su Choi [1,2] and Young Wook Park [1,3,*]

[1] Nano and Organic-Electronics Laboratory, Department of Display and Semiconductor Engineering, Sun Moon University, Asan 31460, Republic of Korea; zeratull1234@sunmoon.ac.kr (T.J.H.); baeejng@korea.ac.kr (E.J.B.); crs4964@korea.ac.kr (G.S.C.)

[2] Display and Nanosystem Laboratory, Department of Electrical Engineering, Korea University, Seoul 02841, Republic of Korea

[3] Center for Next Generation Semiconductor Technology, Department of Display and Semiconductor Engineering, Sun Moon University, Asan 31460, Republic of Korea

* Correspondence: zerook@sunmoon.ac.kr

1. SEM images of the Nanostructures

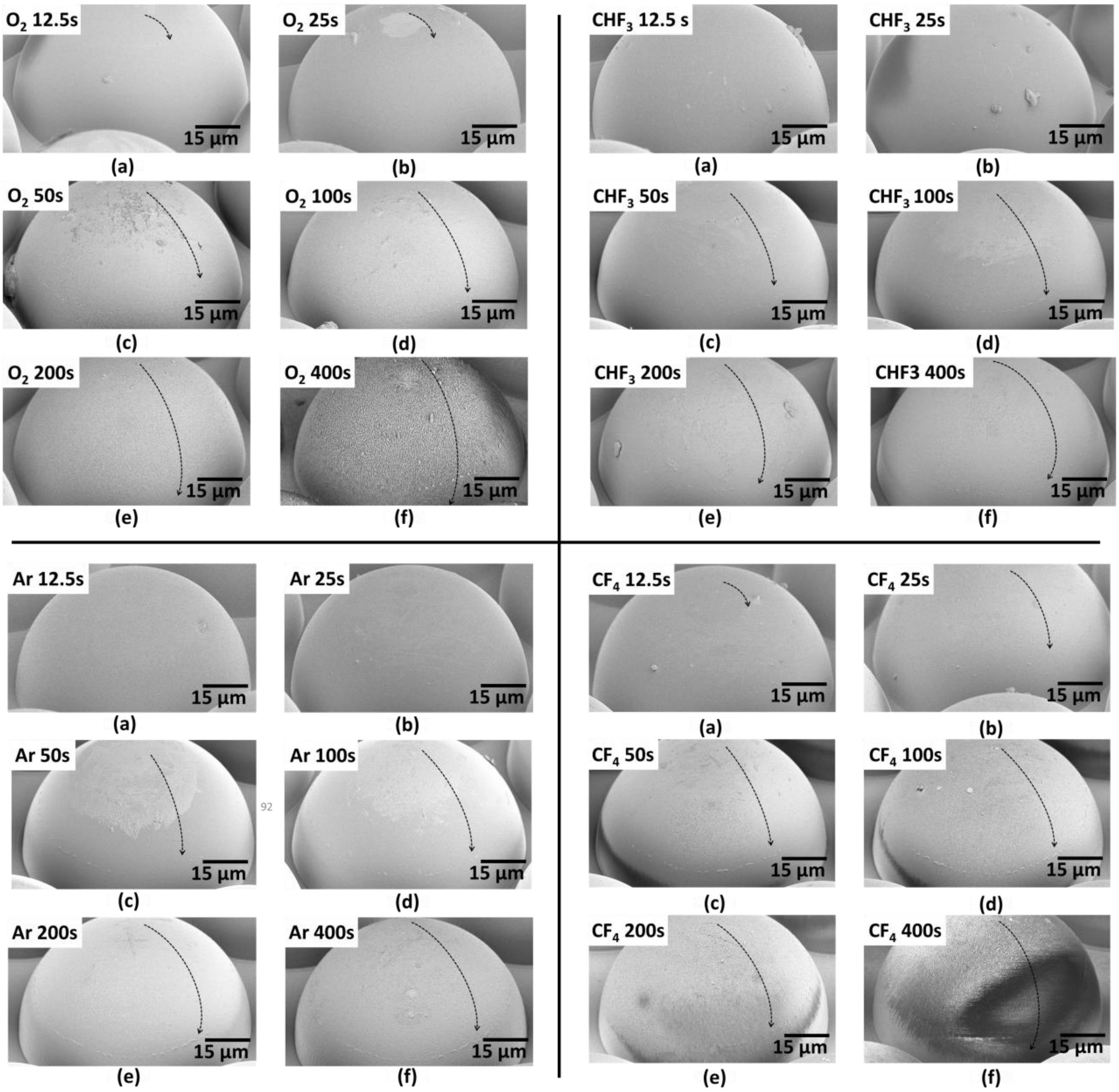

Figure S1. SEM images of the nanostructures formation change by RIE treatment time

Figure S1 shows SEM images for each gas as the RIE treatment time increases. The high aspect ratio of O_2 and CF_4 gas plasma samples gives a distinctive, recognizable difference from the other two gas plasmas (Ar and CHF_3). All the samples show increases in nanostructure coverage following the RIE treatment time increase.

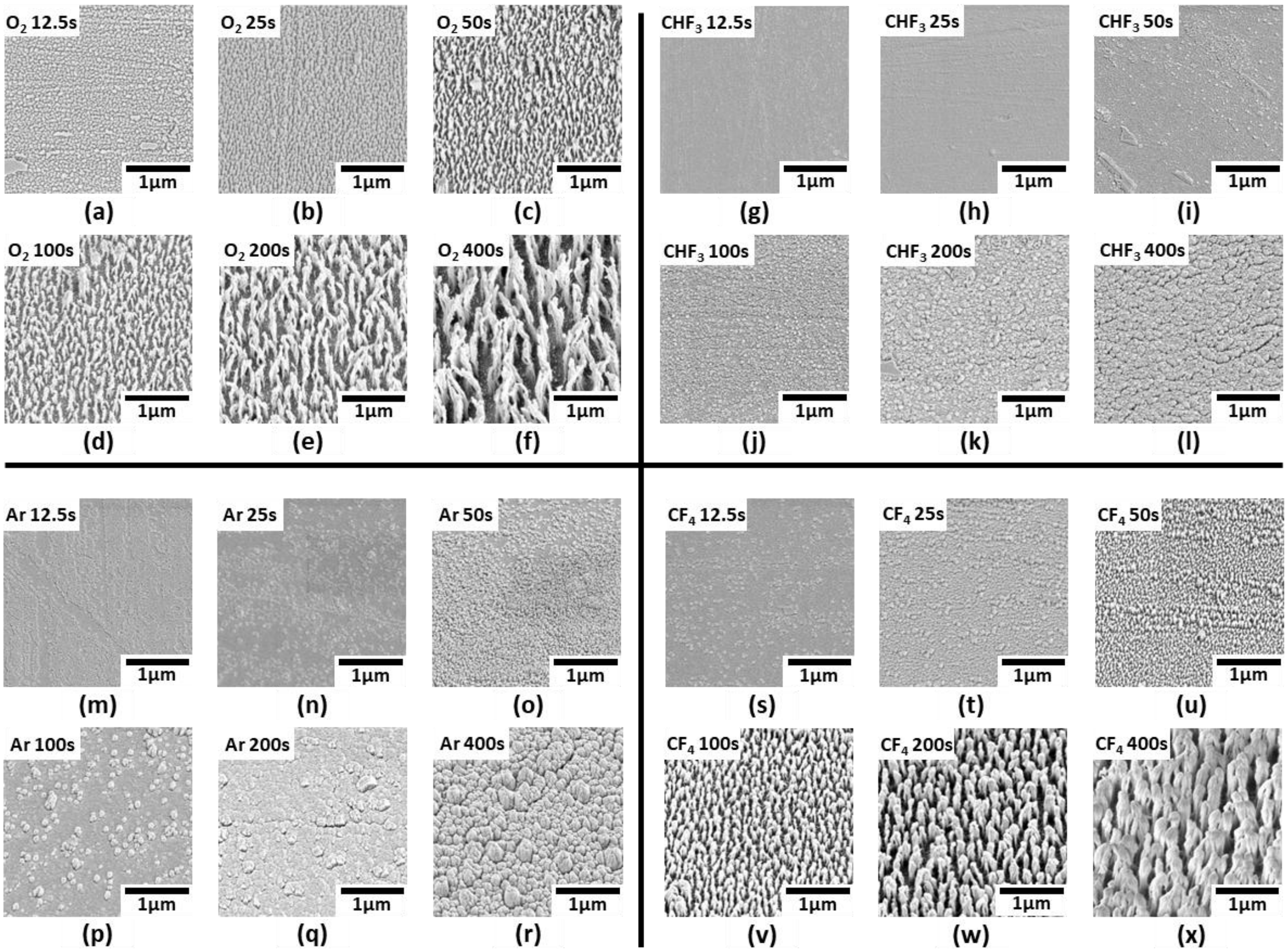

Figure S2. SEM images of the nanostructures fabricated by the different gas plasma

As shown in Figure S2, nanostructures formed by O_2 and CF_4 gas plasmas show high height and aspect ratio, whereas nanopillars formed by CHF_3 and Ar gas plasmas show low aspect ratio.

2. EL characteristics

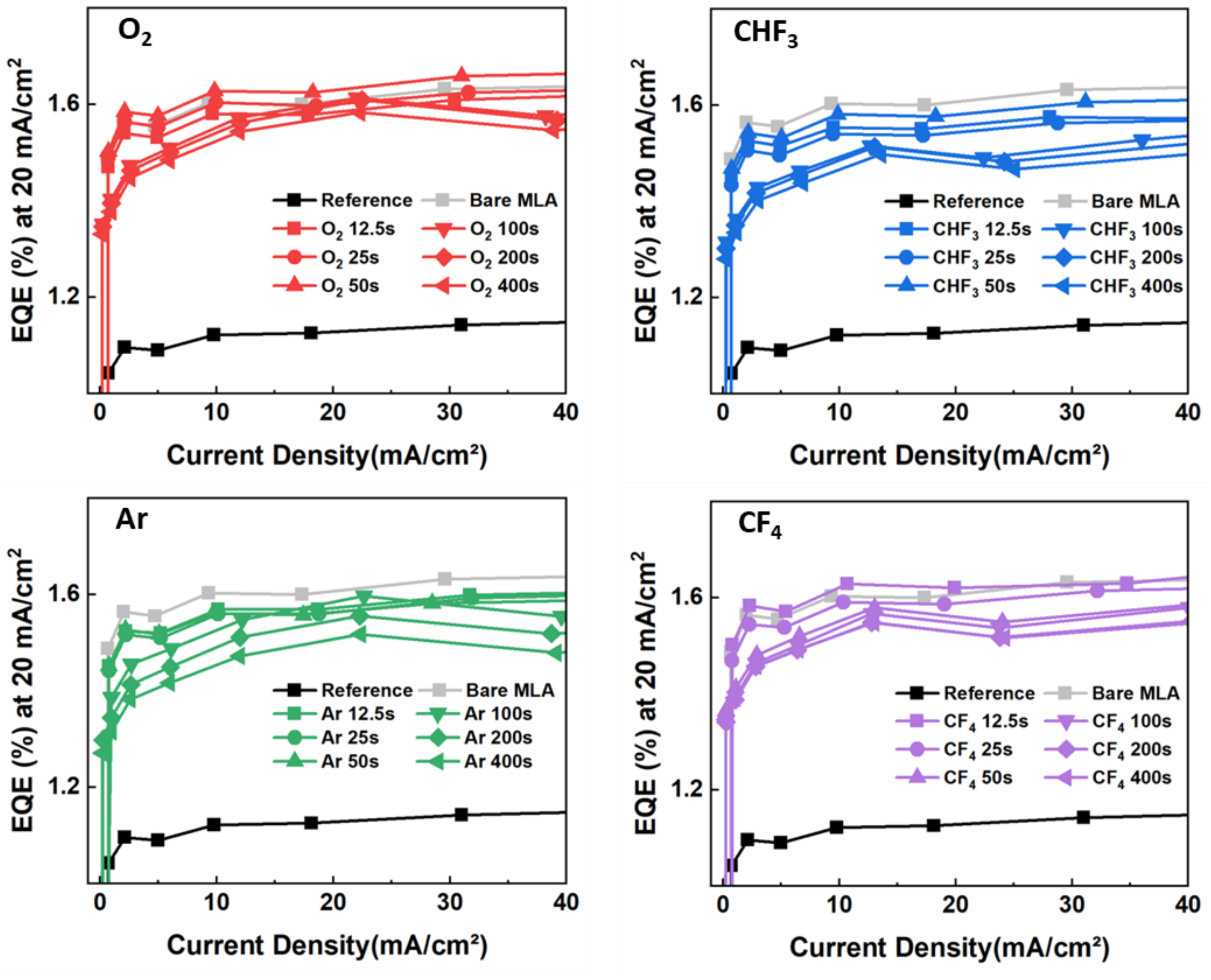

Figure S3. The current density-EQE characteristics

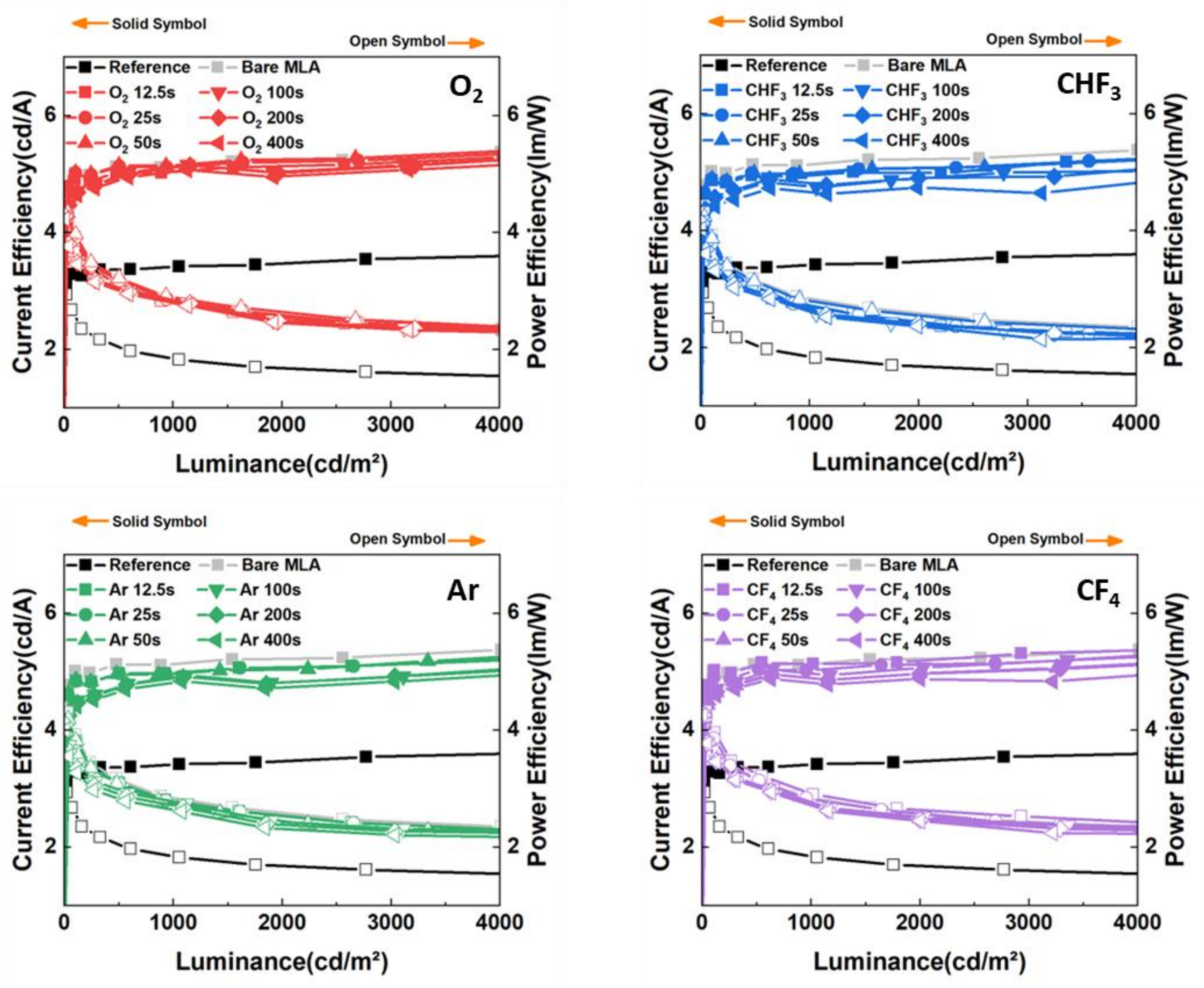

Figure S4. The luminance-current efficiency, luminance-power efficiency characteristics

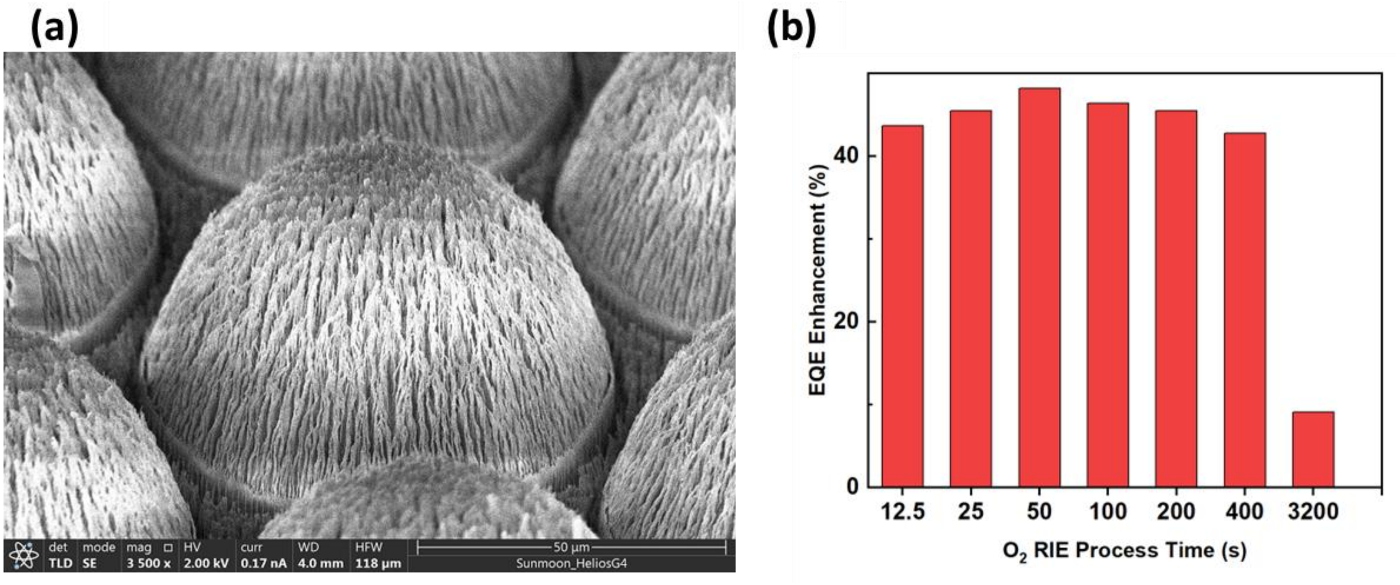

Figure S5. (a) SEM images of the nanostructures formed at 3200 s in O_2 gas plasma (b) EQE Enhancement compared to Reference
